# A Comparative Proteomic Analysis of the Buds and the Young Expanding Leaves of the Tea Plant (*Camellia sinensis* L.)

**DOI:** 10.3390/ijms160614007

**Published:** 2015-06-18

**Authors:** Qin Li, Juan Li, Shuoqian Liu, Jianan Huang, Haiyan Lin, Kunbo Wang, Xiaomei Cheng, Zhonghua Liu

**Affiliations:** 1 Key Laboratory of Tea Science of Ministry of Education, Hunan Agricultural University, Changsha 410128, Hunan, China; E-Mails: liqinvip@126.com (Q.L.); xixi_lj@126.com (J.L.); jian7513@gmail.com (J.H.); xiaomeichengvip@163.com (X.C.); 2 National Research Center of Engineering Technology for Utilization of Functional Ingredients from Botanicals, Hunan Agricultural University, Changsha 410128, Hunan, China; E-Mails: shuoqianliuvip@126.com (S.L.); linhaiyanvip@126.com (H.L.); wkboo163@163.com (K.W.); 3 Collaborative Innovation Centre of Utilisation of Functional Ingredients from Botanicals, Hunan Agricultural University, Changsha 410128, Hunan, China; 4 Hunan Provincial Key Laboratory for Germplasm Innovation and Utilization of Crop, Hunan Agricultural University, Changsha 410128, Hunan, China

**Keywords:** *Camellia sinensis* L., proteome, iTRAQ

## Abstract

Tea (*Camellia sinensis* L.) is a perennial woody plant that is widely cultivated to produce a popular non-alcoholic beverage; this beverage has received much attention due to its pleasant flavor and bioactive ingredients, particularly several important secondary metabolites. Due to the significant changes in the metabolite contents of the buds and the young expanding leaves of tea plants, high-performance liquid chromatography (HPLC) analysis and isobaric tags for relative and absolute quantitation (iTRAQ) analysis were performed. A total of 233 differentially expressed proteins were identified. Among these, 116 proteins were up-regulated and 117 proteins were down-regulated in the young expanding leaves compared with the buds. A large array of diverse functions was revealed, including roles in energy and carbohydrate metabolism, secondary metabolite metabolism, nucleic acid and protein metabolism, and photosynthesis- and defense-related processes. These results suggest that polyphenol biosynthesis- and photosynthesis-related proteins regulate the secondary metabolite content of tea plants. The energy and antioxidant metabolism-related proteins may promote tea leaf development. However, reverse transcription quantitative real-time PCR (RT-qPCR) showed that the protein expression levels were not well correlated with the gene expression levels. These findings improve our understanding of the molecular mechanism of the changes in the metabolite content of the buds and the young expanding leaves of tea plants.

## 1. Introduction

Tea (*Camellia sinensis* L.) is a perennial woody plant that is widely cultivated to produce a popular non-alcoholic beverage; this beverage has received much attention due to its pleasant flavor and bioactive ingredients, particularly several key secondary metabolites [1]. Tea leaves contain important secondary metabolites, including polyphenols (catechins, flavones, anthocyanidin and phenolic acid), alkaloids (theobromine, theophylline and caffeine), and theanine, which not only contribute to tea quality but also have important human health benefits [2].

The changes in the chemical composition of the buds and the young expanding leaves of tea have been extensively studied. A previous study showed that during seeding development, total catechins, epigallocatechin gallate (EGCG) and epicatechin gallate (ECG) decreased, whereas the epigallocatechin (EGC) content increased [3]. As the shoots matured, the total flavonol glycoside and myricetin contents increased, but the kaempferol content decreased [4]. Purine alkaloid metabolism also appears to be closely associated with leaf development and aging in tea seedlings. In addition, the expression levels of several genes related to metabolite synthesis in tea leaves were analyzed. A positive correlation was found between the catechin concentration and the expression of flavanone 3-hydroxylase (F3H) in tea leaves at different developmental stages [5]. A study has shown that most catechins accumulate to higher levels in the shoots than in the mature leaves; similarly, the genes involved in catechin synthesis, including phenylalanine ammonia-lyase 1 (PAL1), chalcone synthase (CHS), dihydroflavonol 4-reductase (DFR), leucoanthocyanidin reductase (LCR), and F3H are more highly expressed in the shoots than in the mature leaves [6]. Zhang *et al.* also found that the content of non-galloylated catechins—except gallocatechin (GC)—as well as the activity of DFR and anthocyanidin reductase (ANR), gradually increased from the buds to the mature leaves [7]. An analysis of purine alkaloids in different parts of the seedlings showed that the caffeine and theobromine content was greater in young leaves and decreased with increasing leaf maturity, and the levels of tea caffeine synthase (TCS) transcripts were also highest in young leaves and declined markedly during leaf development [8,9]. Different levels of metabolites in tea leaves are likely characterized by diverse gene and protein expression profiles at each developmental stage.

Despite studies on the metabolite synthesis-related genes in tea plants, the molecular mechanisms underlying the changes in metabolite content have not yet been examined in detail. In this study, isobaric tags for relative and absolute quantitation (iTRAQ) analysis were first used to separate the differentially expressed proteins. In addition, the content of a set of important metabolites was studied, and the expression of the genes associated with the differentially expressed proteins was also measured. The purpose of this study is to provide an improved understanding of the molecular mechanisms behind the change in the metabolite content between the apical buds and the young expanding leaves of tea plants.

## 2. Results

### 2.1. Analysis of Metabolite Profiles

**Figure 1:**
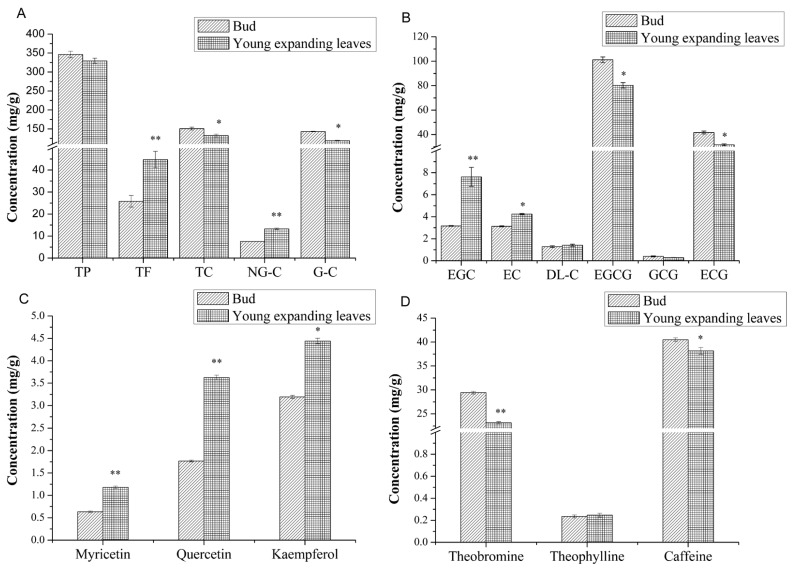
Changes in the levels of secondary metabolites in the buds and the young expanding leaves of tea. (**A**) Total polyphenols (TP), total flavonoids (TF), total catechins (TC), non-galloylated catechins (NG-C) and galloylated catechins (G-C); (**B**) Individual catechins; (**C**) Myicetin, quercetin and kaempferol; and (**D**) Individual alkaloids. Statistical significance: * *p* < 0.05 and ** *p* < 0.01.

To further investigate the important changes in metabolite content, the polyphenol, catechin, and flavonoid contents of the buds and the young expanding leaves of tea plants were analyzed (Figure 1). As shown in Figure 1A, the concentration of total catechin in young expanding leaves (132.507 ± 3.889 mg/g) was 0.839-fold lower (*p* < 0.05) than that in the buds (150.851 ± 3.640 mg/g). The total polyphenol content of the young expanding leaves (329.395 ± 6.984 mg/g) was 0.951-fold lower than that of the buds (346.219 ± 8.609 mg/g), but this difference was not significant (*p* > 0.05). However, the total flavonoid content of the young expanding leaves (44.754 ± 3.731 mg/g) was 1.734-fold higher than that of the buds (25.803 ± 2.619 mg/g) (*p* < 0.01).

The levels of non-galloylated catechins, including EGC, epicatechin (EC) and DL-catechin (DL-C), were significantly greater in the young expanding leaves (13.280 ± 0.338 mg/g) than in the buds (7.574 ± 0.053 mg/g) (*p* < 0.01). However, the contents of galloylated catechins, including EGCG, GCG and ECG, were significantly lower in the young expanding leaves (119.226 ± 0.997 mg/g) than in the buds (143.277 ± 0.823 mg/g) (*p* < 0.05) (Figure 1A). In both the buds and the young expanding leaves the most abundant individual catechin was EGCG, and the least abundant individual catechin was GCG (gallocatechin gallate). The relative concentrations of each individual catechins in both the buds and the young expanding leaves were EGCG > ECG > EGC > EC > DL-C > GCG. The concentrations of EGC and EC in the young expanding leaves (EGC: 7.626 ± 0.859 mg/g, EC: 4.244 ± 0.060 mg/g) were greater than those in the buds (EGC: 3.167 ± 0.034 mg/g, EC: 3.127 ± 0.044 mg/g) (*p* < 0.01 for EGC and *p* < 0.05 for EC), and the level of DL-C was slightly higher (*p* > 0.05) in the young expanding leaves (1.410 ± 0.095 mg/g) than in the buds (1.280 ± 0.081 mg/g). However, the concentrations of EGCG and ECG were lower in the young expanding leaves (EGCG: 80.292 ± 2.216 mg/g, ECG: 38.646 ± 0.769 mg/g) than in the buds (EGCG: 101.169 ± 2.343 mg/g, ECG: 41.705 ± 1.204 mg/g) (*p* < 0.05), and the GCG level was also slightly lower (*p* > 0.05) in the young expanding leaves (0.288 ± 0.008 mg/g) than in the buds (0.403 ± 0.051 mg/g) (Figure 1B). In the young expanding leaves, the levels of individual flavonols, including myricetin, quercetin and kaempferol, were all greater than those in the buds (1.181 ± 0.026 mg/g myricetin, 3.627 ± 0.051 mg/g quercetin, and 4.441 ± 0.063 mg/g kaempferol in the leaves compared with 0.635 ± 0.017 mg/g myricetin, 1.767 ± 0.021 mg/g quercetin, and 3.193 ± 0.038 mg/g kaempferol in the buds, *p* < 0.01 for myricetin and quercetin, and *p* < 0.05 for kaempferol) (Figure 1C). Three types of alkaloids, including theobromine, theophylline and caffeine, were also detected via HPLC analysis. The theobromine and caffeine levels were lower in the young expanding leaves than in the buds (theobromine: 23.165 ± 0.213 mg/g in leaves and 29.418 ± 0.299 mg/g in buds, *p* < 0.01; caffeine: 38.167 ± 0.704 mg/g in leaves and 40.484 ± 0.396 mg/g in buds, *p* < 0.05), and the theophylline levels were slightly higher in the young expanding leaves (0.247 ± 0.017 mg/g) compared with the buds (0.235 ± 0.013 mg/g) (*p* > 0.05) (Figure 1D). Due to the significant changes in the metabolite contents of the buds and the young expanding leaves of tea plants, iTRAQ analysis was performed to determine the molecular mechanisms behind this change.

### 2.2. Protein Identification

**Figure 2:**
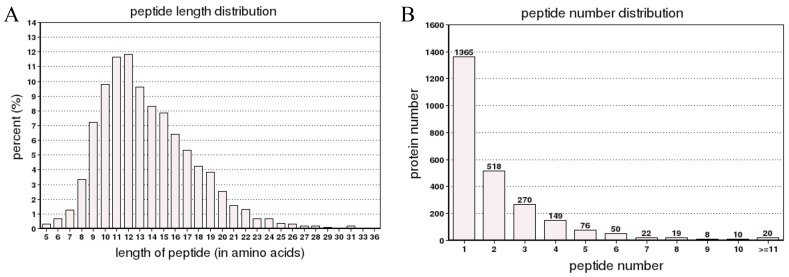
The spectra, peptides, and proteins, as well as the number of peptides in the iTRAQ proteomic analysis identified as matching proteins. The spectra, peptides and proteins were identified by searching against a database (**A**); and The number of peptides matched to proteins using MASCOT (**B**).

To explore the correlation between the proteomic and metabolite profiles of buds and young expanding leaves, samples were analyzed by iTRAQ proteomics coupled with LC-MS/MS. A total of 60,820 spectra were generated from the iTRAQ experiment and the data were analyzed using Mascot software. A total of 8015 spectra were matched to known spectra, 6974 spectra were matched to unique spectra, 4746 were matched to peptides, 4260 were matched to unique peptides and 2507 were matched to proteins (Figure 2A). The distribution of the number of peptides defining each protein is shown in Figure 2B; over 55% of the proteins were represented by at least two peptides.

### 2.3. Functional Classification of the Differentially Expressed Proteins

The proteins whose levels changed more than 1.5-fold and had a *p*-values of less than 0.05 were considered differentially expressed. Based on these two criteria, 233 proteins were differentially expressed between the buds and the young expanding leaves, and these proteins were isolated and quantified using comparative proteomics via iTRAQ. Of the 233 differentially expressed proteins, 116 were more abundant and 117 were less abundant in the young expanding leaves compared with the buds. GO analysis revealed that the differentially expressed proteins participated in several biological processes (*p* < 0.05), as shown in Table S1. KEGG enrichment analysis suggested that the differentially expressed proteins are involved in several pathways (*p* < 0.05), including phenylalanine metabolism (Table S2).

**Figure 3:**
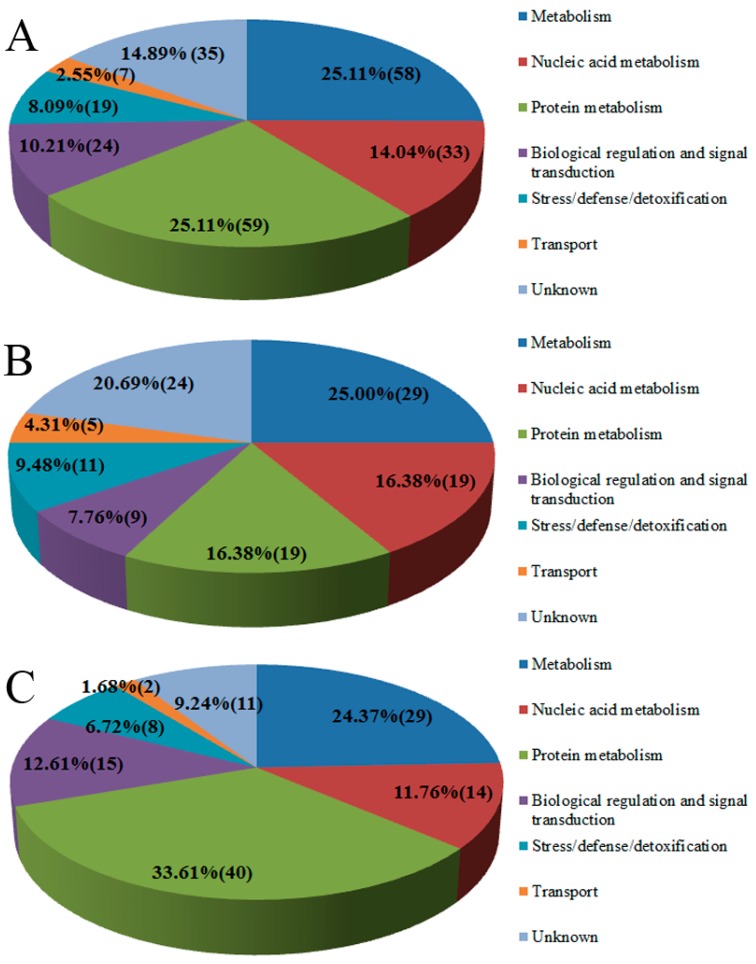
Functional classification of the differentially expressed proteins. Functional groups and the numbers of proteins of all 233 differentially expressed proteins that fall into each group (**A**); categorization of the 116 up-regulated proteins (**B**); and categorization of the 117 down-regulated proteins (**C**). The number in each functional category represents the number of proteins in that category.

The proteins were classified into seven functional categories based on their functional biological properties and pathways: metabolism (58, 25.11%), nucleic acid metabolism (33, 14.04%), protein metabolism (59, 25.11%), biological regulation and signal transduction (24, 10.21%), stress/defense/detoxification (19, 8.09%), transport (7, 2.55%), and unknown function (35, 14.89%) (Figure 3A). Of the up-regulated proteins, 25.00% (29 proteins) function in metabolism, 16.38% (19 proteins) function in nucleic acid metabolism, 16.38% (19 proteins) are involved in protein metabolism, 7.76% (nine proteins) have biological regulation and signal transduction function, 9.58% (11 proteins) function in stress/defense/detoxification, 4.31% (5 proteins) are involved in transport and 20.69% of them (24 proteins) were of unknown function (Figure 3B). Among the down-regulated proteins, 24.37% (29 proteins) function in metabolism, 11.76% (14 proteins) function in nucleic acid metabolism, 33.61% (40 proteins) have a role in protein metabolism, 12.61% (15 proteins) are involved in biological regulation and signal transduction, 6.72% (8 proteins) are involved in stress/defense/detoxification, 1.68% (two proteins) function in transport and 9.24% (11 proteins) were of unknown function (Figure 3C). More detailed information can be found in Table 1.

**Table 1:** List of proteins that are differentially expressed between the buds and the young expanding leaves of tea plants.

Accession Number	Proteins Name and Species	Score	Mass (Da)	Coverage	Peptide Count	Fold Change (Leaves/Bud)	Function
gi|350536667|	Dihydrolipoamide dehydrogenase precursor [*Solanum lycopersicum*]	202	68,166	13.9	5	2.164	Metabolism
gi|15081610|	Xyloglucan endotransglycosylase XET2 [*Vitis vinifera*]	137	39,952	12.4	3	1.614	Metabolism
gi|76786311|	Flavonol synthase [*Camellia sinensis*]	288	45,768	27.9	6	1.788	Metabolism
gi|225458243|	PREDICTED: isoflavone reductase homolog P3 [*Vitis vinifera*]	315	37,529	31.6	7	2.649	Metabolism
gi|359491464|	PREDICTED: lysosomal α-mannosidase [*Vitis vinifera*]	111	38,176	12.7	3	1.825	Metabolism
gi|71535021|	α-glucosidase [*Medicago sativa*]	204	86,320	6	4	2.353	Metabolism
gi|255578100|	Dihydrolipoamide succinyltransferase component of 2-oxoglutarate dehydrogenase, putative [*Ricinus communis*]	57	38,166	6.9	2	1.878	Metabolism
gi|225426623|	PREDICTED: 2-keto-3-deoxy-l-rhamnonate aldolase-like [*Vitis vinifera*]	78	21,742	10.2	1	2.281	Metabolism
gi|193290728|	Putative pyruvate dehydrogenase E3 subunit [*Capsicum annuum*]	74	42,069	8.3	2	1.567	Metabolism
gi|255566959|	NADH-cytochrome B5 reductase, putative [*Ricinus communis*]	32	11,336	11.4	1	1.58	Metabolism
sp|Q9SVG4|	Reticuline oxidase-like protein [*Spinacia oleracea*]	108	30,376	6.9	1	1.729	Metabolism
sp|Q9M069|	Glucan endo-1,3-β-glucosidase 7 [*Arabidopsis thaliana*]	139	40,171	16.2	3	1.568	Metabolism
gi|147767550|	Hypothetical protein VITISV_013343 [*Vitis vinifera*]	159	20,700	22.4	2	1.542	Metabolism
gi|193290702|	Putative 3-isopropylmalate dehydrogenase small subunit [*Capsicum annuum*]	354	25,595	36.2	4	1.681	Metabolism
gi|225451235|	PREDICTED: cysteine synthase isoform 2 [*Vitis vinifera*]	321	47,206	14.9	4	2.216	Metabolism
gi|225454278|	PREDICTED: cysteine synthase, chloroplastic/chromoplastic isoform 1 [*Vitis vinifera*]	60	40,113	10.5	2	1.905	Metabolism
sp|P50246|	Adenosylhomocysteinase [*Medicago sativa*]	186	65,326	11.9	5	1.551	Metabolism
sp|P47999|	Cysteine synthase, chloroplastic/chromoplastic [*Arabidopsis thaliana*]	111	51,056	14.5	4	1.856	Metabolism
sp|P94170|	Carbonic anhydrase [*Nostoc* sp.]	191	12,938	27.6	2	2.527	Metabolism
sp|Q42876|	Leucine aminopeptidase 2, chloroplastic [*Solanum lycopersicum*]	124	74,254	3.9	2	1.852	Metabolism
gi|75755999|	TO87b-13 [*Taraxacum officinale*]	260	83,317	6	3	2.18	Metabolism
gi|330318698|	Light-inducible protein atls1 [*Camellia sinensis*]	115	18,370	8.8	1	2.68	Metabolism
gi|224098154|	predicted protein [*Populus trichocarpa*]	145	29,956	28.8	5	1.504	Metabolism
gi|225433426|	PREDICTED: 3-ketoacyl-CoA thiolase 2, peroxisomal isoform 2 [*Vitis vinifera*]	197	7506	51.9	2	1.568	Metabolism
gi|225462452|	PREDICTED: GDSL esterase/lipase At5g45670 [*Vitis vinifera*]	250	22,819	30.5	4	1.79	Metabolism
sp|Q93YW8|	GDSL esterase/lipase At4g18970 [*Arabidopsis thaliana*]	250	22,819	30.5	4	1.79	Metabolism
sp|Q9SYF0|	GDSL esterase/lipase 2 [*Arabidopsis thaliana*]	316	46,565	14.2	5	2.203	Metabolism
sp|Q9LZS7|	GDSL esterase/lipase At5g03610 [*Arabidopsis thaliana*]	80	45,316	11.8	3	3.399	Metabolism
gi|296088201|	Unnamed protein product [*Vitis vinifera*]	73	30,392	9.5	2	1.55	Metabolism
gi|18140567|	Ribulose-1,5-bisphosphate carboxylase/oxygenase large subunit [*Camellia japonica*]	518	52,867	15.8	6	0.357	Metabolism
gi|156106226|	Rubisco activase [*Camellia sinensis*]	646	43,205	31.4	7	0.564	Metabolism
gi|20257362|	Ribulose 1,5-bisphosphate carboxylase/oxygenase, partial (chloroplast) [*Schima superba*]	303	24,090	10.3	2	0.465	Metabolism
gi|255553993|	Phosphoenolpyruvate carboxylase, putative [*Ricinus communis*]	108	32,666	14.7	3	0.469	Metabolism
gi|169807676|	NADP-dependent glyceraldehyde-3-phosphate dehydrogenase [*Platanus x acerifolia*]	322	69,081	17	6	0.543	Metabolism
gi|356524319|	PREDICTED: probable glycerophosphoryl diester phosphodiesterase 1-like [*Glycine max*]	212	56,841	8.8	3	0.552	Metabolism
gi|2266947|	Phosphoenolpyruvate carboxylase 1 [*Gossypium hirsutum*]	173	94,629	7.7	5	0.477	Metabolism
gi|255581778|	chlorophyll A/B binding protein, putative [*Ricinus communis*]	96	41,261	3.8	1	0.664	Metabolism
sp|P81833|	Thylakoid lumenal 29 kDa protein, chloroplastic (Fragment) [*Spinacia oleracea*]	176	43,057	22.2	4	0.651	Metabolism
sp|Q8H1Q1|	Thylakoid lumenal protein At1g12250, chloroplastic [*Spinacia oleracea*]	254	35,331	21.5	4	0.655	Metabolism
sp|O04138|	Chitinase 4 [*Oryza sativa* subsp. *Japonica*]	223	30,457	19.4	3	0.466	Metabolism
sp|Q9FKK7|	Xylose isomerase [*Spinacia oleracea*]	172	63,511	13	4	0.575	Metabolism
gi|27804768|	Sedoheptulose-1,7-bisphosphatase precursor [*Oryza sativa Indica* Group]	268	54,158	8.7	3	0.58	Metabolism
gi|380508822|	Putative hydroxycinnamoyl-CoA:shikimate/quinate hydroxycinnamoyltransferase [*Camellia sinensis*]	48	7531	32.7	2	0.493	Metabolism
gi|330318804|	Photosystem I reaction center subunit XI [*Camellia sinensis*]	132	27,128	20.5	3	0.63	Metabolism
gi|357521691|	Atypical receptor-like kinase MARK [*Medicago truncatula*]	94	45,630	9.6	3	0.562	Metabolism
gi|357494517|	Calcium dependent protein kinase [*Medicago truncatula*]	118	14,910	21.8	2	0.422	Metabolism
gi|297744280|	Unnamed protein product [*Vitis vinifera*]	82	49,263	13.9	3	0.47	Metabolism
sp|Q56YA5|	Serine--glyoxylate aminotransferase [*Spinacia oleracea*]	124	32,097	7	1	0.251	Metabolism
sp|P45726|	Phenylalanine ammonia-lyase [*Camellia sinensis*]	463	90,257	19.2	11	0.63	Metabolism
gi|71480741|	β-1,3-glucanase [*Camellia sinensis*]	126	60,244	2.4	1	0.565	Metabolism
sp|P46637|	Arginase [*Spinacia oleracea*]	470	39,198	21	6	0.47	Metabolism
sp|Q6AUR2|	Nitrogen regulatory protein P-II homolog [*Oryza sativa* subsp. *Japonica*]	101	28,420	12.8	3	0.648	Metabolism
gi|302566881|	Lipoxygenase [*Camellia sinensis*]	72	59,000	10.2	3	0.54	Metabolism
gi|194466253|	*N*-acetyltransferase [*Arachis hypogaea*]	103	29,065	12	2	0.605	Metabolism
sp|Q9LZ72|	3-ketoacyl-CoA synthase 21 [*Spinacia oleracea*]	120	62,270	7.7	3	0.532	Metabolism
sp|Q570B4|	3-ketoacyl-CoA synthase 10 [*Spinacia oleracea*]	53	38,242	3.8	1	0.455	Metabolism
sp|Q9FJ41|	GDSL esterase/lipase At5g45950 [*Spinacia oleracea*]	138	50,893	9.3	2	0.619	Metabolism
sp|Q9LEB4|	Polyadenylate-binding protein RBP45 [*Nicotiana plumbaginifolia*]	135	56,285	10.6	4	1.53	Nucleic acid metabolism
sp|Q43349|	29 kDa ribonucleoprotein, chloroplastic [*Spinacia oleracea*]	43	34,389	7.1	2	1.624	Nucleic acid metabolism
sp|P43333|	U2 small nuclear ribonucleoprotein A´ [*Spinacia oleracea*]	135	40,620	14.3	3	1.574	Nucleic acid metabolism
gi|307940738|	G-strand specific single-stranded telomere-binding protein 1 [*Nicotiana tabacum*]	250	22,819	30.5	4	1.79	Nucleic acid metabolism
sp|Q84L31|	Putative DNA repair protein RAD23-3 [*Spinacia oleracea*]	174	42,346	15.4	4	1.533	Nucleic acid metabolism
gi|255603771|	DNA binding protein, putative [*Ricinus communis*]	250	22,819	30.5	4	1.79	Nucleic acid metabolism
gi|255603771|	DNA binding protein, putative [*Ricinus communis*]	177	41,602	16.9	3	2.491	Nucleic acid metabolism
sp|Q9S7C9|	Putative DNA-binding protein ESCAROLA [*Spinacia oleracea*]	250	38,185	17	4	1.664	Nucleic acid metabolism
gi|79596510|	AT hook motif DNA-binding family protein [*Spinacia oleracea*]	137	29,457	21.1	4	2.576	Nucleic acid metabolism
sp|Q9S7C9|	Putative DNA-binding protein ESCAROLA [*Spinacia oleracea*]	250	38,185	17	4	1.664	Nucleic acid metabolism
gi|45533923|	Glycine-rich RNA-binding protein RGP-1c [Nicotiana sylvestris]	487	21,422	28.1	4	2.945	Nucleic acid metabolism
sp|Q9SVM8|	Glycine-rich RNA-binding protein 2, mitochondrial [*Spinacia oleracea*]	410	20,091	17.1	2	1.506	Nucleic acid metabolism
gi|225440996|	PREDICTED: histone deacetylase HDT1-like [*Vitis vinifera*]	99	37,638	12.2	3	3.522	Nucleic acid metabolism
sp|Q9XI36|	Methyl-CpG-binding domain-containing protein 10 [*Spinacia oleracea*]	285	42,453	37.6	7	1.364	Nucleic acid metabolism
gi|225457458|	PREDICTED: transcription factor BTF3 [*Vitis vinifera*]	170	25,374	35.6	4	1.829	Nucleic acid metabolism
gi|297723091|	Os04g0385700 [*Oryza sativa Japonica* Group]	56	34,525	4.3	1	2.34	Nucleic acid metabolism
gi|296081863|	Unnamed protein product [*Vitis vinifera*]	177	38,697	15.7	3	2.094	Nucleic acid metabolism
gi|297744195|	Unnamed protein product [*Vitis vinifera*]	155	29,045	22.4	3	2.146	Nucleic acid metabolism
gi|255642098|	Unknown [*Glycine max*]	119	51,914	10.3	4	2.23	Nucleic acid metabolism
sp|Q9LFN6|	DEAD-box ATP-dependent RNA helicase 56 [*Spinacia oleracea*]	220	54,506	18.6	5	0.649	Nucleic acid metabolism
sp|Q84UQ1|	DEAD-box ATP-dependent RNA helicase 42 [*Oryza sativa* subsp. *Japonica*]	98	120,162	2.2	2	0.325	Nucleic acid metabolism
sp|B6EUA9|	Pre-mRNA-processing protein 40A [*Spinacia oleracea*]	242	83,063	9.7	5	0.233	Nucleic acid metabolism
sp|O22315|	Pre-mRNA-splicing factor SF2 [*Spinacia oleracea*]	126	7308	35.7	2	0.432	Nucleic acid metabolism
gi|374095609|	Spliceosomal-like protein [*Camellia sinensis*]	23	4551	17.1	1	0.385	Nucleic acid metabolism
sp|Q9S709|	Splicing factor U2af small subunit A [*Spinacia oleracea*]	71	29,349	7.9	1	0.637	Nucleic acid metabolism
sp|P81766|	Nucleoside diphosphate kinase 3 [*Spinacia oleracea*]	61	31,625	7.5	2	0.508	Nucleic acid metabolism
gi|224117596|	Predicted protein [*Populus trichocarpa*]	368	54,505	13.4	5	0.398	Nucleic acid metabolism
gi|225462994|	PREDICTED: DNA replication licensing factor mcm5-A-like [*Vitis vinifera*]	259	94,881	13.6	8	0.582	Nucleic acid metabolism
sp|O04716|	DNA mismatch repair protein MSH6 [*Spinacia oleracea*]	159	50,441	4.6	1	0.276	Nucleic acid metabolism
gi|359386142|	RNA recognition motif protein 1 [Citrus sinensis]	155	14,712	42.7	3	0.492	Nucleic acid metabolism
gi|195626496|	Glycine-rich RNA-binding protein 2 [*Zea mays*]	318	21,175	33.1	4	0.435	Nucleic acid metabolism
sp|Q9FLH0|	PUTATIVE nuclear matrix constituent protein 1-like protein [*Spinacia oleracea*]	69	78,347	5.6	2	0.66	Nucleic acid metabolism
gi|385213056|	20S proteasome β2 subunit, partial [Oryza brachyantha]	163	40,674	14.1	4	2.297	Protein metabolism
gi|49175785|	26S proteasome β subunit [*Pisum sativum*]	187	35,781	16	4	1.632	Protein metabolism
gi|16225442|	26S proteasome regulatory subunit S12 isolog-like protein [Castanea sativa]	144	38,542	10.6	3	2.263	Protein metabolism
gi|225431100|	PREDICTED: 26S proteasome non-ATPase regulatory subunit 4 [*Vitis vinifera*]	73	8946	16.9	1	1.553	Protein metabolism
gi|24473796|	60s acidic ribosomal protein [*Prunus dulcis*]	208	14,983	15.8	2	2.924	Protein metabolism
gi|330318716|	60S acidic ribosomal protein p2 [*Camellia sinensis*]	156	15,062	16.2	2	4.223	Protein metabolism
sp|Q8LEQ0|	60S acidic ribosomal protein P1-3 [*Spinacia oleracea*]	185	19,758	10.1	1	1.928	Protein metabolism
sp|Q9SVZ6|	60S acidic ribosomal protein P3-1 [*Spinacia oleracea*]	666	15,391	13.7	1	1.866	Protein metabolism
gi|255574159|	Proteasome subunit β type 6,9, putative [*Ricinus communis*]	368	31,544	22.1	5	1.644	Protein metabolism
gi|255564428|	Elongation factor 1-β, putative [*Ricinus communis*]	62	33,531	5.7	1	1.899	Protein metabolism
gi|255539639|	Cucumisin precursor, putative [*Ricinus communis*]	86	56,932	2.8	1	1.535	Protein metabolism
gi|14594919|	Putative α5 proteasome subunit [*Nicotiana tabacum*]	170	30,889	13.3	3	2.021	Protein metabolism
gi|356549495|	PREDICTED: heat shock 70 kDa protein, mitochondrial-like [*Glycine max*]	62	12,864	10.9	1	1.605	Protein metabolism
gi|272716096|	Disulfide isomerase-like protein [*Gloeospermum blakeanum*]	87	43,279	12.1	2	1.781	Protein metabolism
gi|272716065|	Disulfide isomerase [*Gloeospermum blakeanum*]	250	22,819	30.5	4	1.79	Protein metabolism
sp|Q8VX13|	Protein disulfide isomerase-like 1-3 [*Spinacia oleracea*]	165	80,245	10.1	5	1.625	Protein metabolism
sp|O65351|	SUBTILISIN-like protease [*Spinacia oleracea*]	128	32,198	16.7	3	1.556	Protein metabolism
gi|359473000|	PREDICTED: aspartic proteinase nepenthesin-1-like [*Vitis vinifera*]	250	22,819	30.5	4	1.79	Protein metabolism
sp|P81898|	Peptide-N4-( *N*-acetyl-β-glucosaminyl)asparagine amidase A [*Prunus dulcis*]	49	28,569	5.3	1	1.823	Protein metabolism
gi|7141245|	26S proteasome regulatory ATPase subunit S10b [*Vitis riparia*]	164	54,246	13.8	4	0.539	Protein metabolism
gi|56481167|	40S ribosomal protein S3a [*Pseudotsuga menziesii* var. *menziesii*]	119	40,753	17.6	3	0.437	Protein metabolism
sp|Q9SCM3|	40S ribosomal protein S2-4 [*Spinacia oleracea*]	253	38,711	12.9	3	0.548	Protein metabolism
gi|241865275|	40S RPS3B [*Sonneratia alba*]	150	30,886	27.4	5	0.536	Protein metabolism
gi|255569736|	40S ribosomal protein S6, putative [*Ricinus communis*]	88	41,992	8.8	2	0.574	Protein metabolism
gi|330318726|	40S ribosomal protein s9 [*Camellia sinensis*]	126	28,301	14	3	0.513	Protein metabolism
gi|357444481|	40S ribosomal protein S18 [*Medicago truncatula*]	223	25,387	24.2	3	0.414	Protein metabolism
gi|255544840|	40S ribosomal protein S2, putative [*Ricinus communis*]	202	35,538	13.4	3	0.64	Protein metabolism
gi|255549228|	40S ribosomal protein S4, putative [*Ricinus communis*]	277	39,687	25.6	6	0.415	Protein metabolism
gi|241865275|	40S RPS3B [*Sonneratia alba*]	209	31,709	27.4	5	0.538	Protein metabolism
sp|Q9ZNS1|	40S ribosomal protein S7 [*Avicennia marina*]	58	32,180	12.8	3	0.61	Protein metabolism
sp|O80360|	50S ribosomal protein L3, chloroplastic (Fragment) [*Nicotiana tabacum*]	179	36,343	19.5	4	0.591	Protein metabolism
gi|255551787|	60S ribosomal protein L22, putative [*Ricinus communis*]	119	22,528	22.1	3	0.615	Protein metabolism
gi|148466442|	60S ribosomal protein L21 [Paeonia suffruticosa]	56	26,661	9.8	2	0.467	Protein metabolism
sp|P51413|	60S ribosomal protein L17-2 [*Spinacia oleracea*]	48	28,359	5.1	1	0.525	Protein metabolism
sp|Q6UNT2|	60S ribosomal protein L5 [*Cucumis sativus*]	90	44,735	6.7	2	0.661	Protein metabolism
sp|Q9SPB3|	60S ribosomal protein L10 [*Vitis riparia*]	189	33,718	12.9	3	0.532	Protein metabolism
sp|P30707|	60S ribosomal protein L9 [*Pisum sativum*]	184	33,248	26.4	4	0.641	Protein metabolism
gi|225427377|	PREDICTED: 60S ribosomal protein L37a-like [*Vitis vinifera*]	93	15,986	16.3	1	0.64	Protein metabolism
gi|330318574|	Ribosomal petrp-like protein [*Camellia sinensis*]	48	28,359	5.1	1	0.525	Protein metabolism
gi|3885519|	Similar to ribosomal protein L32 [*Medicago sativa*]	86	23,581	13.9	2	0.363	Protein metabolism
gi|209922600|	Elongation factor 1-α [Prunus persica]	351	80,605	24.2	11	0.624	Protein metabolism
gi|225452282|	PREDICTED: elongation factor Tu, chloroplastic-like isoform 1 [*Vitis vinifera*]	313	57,834	26.6	8	0.593	Protein metabolism
gi|356524672|	PREDICTED: eukaryotic translation initiation factor 3 subunit C-like [*Glycine max*]	58	6862	27.3	1	0.545	Protein metabolism
gi|71534902|	Histidyl-tRNA synthetase [*Medicago sativa*]	71	41,277	10.8	2	0.603	Protein metabolism
sp|P31542|	ATP-dependent Clp protease ATP-binding subunit clpA homolog CD4B, chloroplastic [*Solanum lycopersicum*]	497	55,091	24.1	8	0.562	Protein metabolism
gi|356516495|	PREDICTED: chaperone protein ClpC, chloroplastic-like [*Glycine max*]	497	55,091	24.1	8	0.562	Protein metabolism
gi|52075839|	Putative chloroplast protease [*Oryza sativa Japonica* Group]	340	85,534	15.4	8	0.523	Protein metabolism
sp|Q8VY06|	Presequence protease 2, chloroplastic/mitochondrial [*Spinacia oleracea*]	85	35,368	13.8	3	0.622	Protein metabolism
sp|Q75GT3|	Chaperone protein ClpB2, chloroplastic [*Oryza sativa* subsp. *Japonica*]	385	130,278	16.2	11	0.504	Protein metabolism
gi|225431090|	PREDICTED: proteasome subunit α type-7 [*Vitis vinifera*]	292	35,407	21.6	4	0.646	Protein metabolism
gi|225457058|	PREDICTED: T-complex protein 1 subunit gamma [*Vitis vinifera*]	354	76,271	13.4	6	0.552	Protein metabolism
gi|225459806|	PREDICTED: T-complex protein 1 subunit β [*Vitis vinifera*]	975	60,327	38.8	11	0.482	Protein metabolism
gi|255567297|	chaperonin containing t-complex protein 1, α subunit, tcpa, putative [*Ricinus communis*]	84	28,459	18.8	3	0.622	Protein metabolism
sp|P32955|	Cysteine proteinase 2 (Fragment) [*Carica candamarcensis*]	385	130,278	16.2	11	0.504	Protein metabolism
sp|P35016|	Endoplasmin homolog [*Catharanthus roseus*]	416	123,589	18.5	13	0.657	Protein metabolism
sp|P38661|	Probable protein disulfide-isomerase A6 [*Medicago sativa*]	213	54,232	24.9	8	0.665	Protein metabolism
sp|Q5Z974|	ATP-dependent zinc metalloprotease FTSH 1, chloroplastic [*Oryza sativa* subsp. *Japonica*]	281	42,007	19.7	4	0.427	Protein metabolism
gi|147766666|	Hypothetical protein VITISV_035841 [*Vitis vinifera*]	177	44,924	17.6	4	0.557	Protein metabolism
gi|224141163|	Predicted protein [*Populus trichocarpa*]	60	36,379	10.7	2	0.56	Protein metabolism
gi|59797458|	Superoxide dismutase [*Lilium hybrid cultivar*]	223	21,087	29.1	3	1.849	Stress/defense/detoxification
sp|Q93VQ9|	Thioredoxin O2, mitochondrial [*Spinacia oleracea*]	80	25,925	12	2	1.907	Stress/defense/detoxification
gi|536838|	NADPH thioredoxin reductase, partial [*Helianthus annuus*]	207	45,192	17.4	4	1.778	Stress/defense/detoxification
sp|Q9LS40|	protein aspartic protease in guard cell 1 [*Rabidopsis thaliana*]	193	48,663	17.6	5	1.635	Stress/defense/detoxification
sp|Q96520|	Peroxidase 12 [*Spinacia oleracea*]	132	41,132	15.2	3	1.899	Stress/defense/detoxification
gi|3201547|	Endochitinase [*Persea americana*]	79	18,633	4.3	1	1.971	Stress/defense/detoxification
sp|Q06015|	Endochitinase 3 (Fragment) [*Arachis hypogaea*]	167	39,946	13.3	3	1.691	Stress/defense/detoxification
gi|215398978|	Dehydrin [*Camellia sinensis*]	44	20,578	11	2	5.811	Stress/defense/detoxification
gi|15637350|	Glutaredoxin [*Tilia platyphyllos*]	150	18,171	10.9	1	1.74	Stress/defense/detoxification
sp|P13240|	Disease resistance response protein 206 [*Pisum sativum*]	167	39,946	13.3	3	1.691	Stress/defense/detoxification
sp|O80934|	Uncharacterized protein At2g37660, chloroplastic [*rabidopsis thaliana*]	161	35,389	21.8	4	1.963	Stress/defense/detoxification
gi|75138338|	Peroxiredoxin Q, chloroplastic [*Gentiana triflora*]	95	27,886	11.1	3	0.595	Stress/defense/detoxification
sp|O23044|	Peroxidase 3 [*Spinacia oleracea*]	241	36,174	19.8	5	0.584	Stress/defense/detoxification
sp|A7NY33|	Peroxidase 4 [*Vitis vinifera*]	119	33,610	21.9	4	0.599	Stress/defense/detoxification
sp|P22242|	Desiccation-related protein PCC13-62 [*Craterostigma plantagineum*]	695	21,349	33.3	4	0.615	Stress/defense/detoxification
gi|270064305|	Abscisic stress ripening [Musa ABB Group]	243	26,152	15.3	2	0.265	Stress/defense/detoxification
sp|Q41328|	Pto-interacting protein 1 [*Solanum lycopersicum*]	52	42,570	13.3	3	0.63	Stress/defense/detoxification
sp|Q9FM19|	Hypersensitive-induced response protein 1 [*Spinacia oleracea*]	124	37,917	9.1	2	0.517	Stress/defense/detoxification
sp|P85524|	kirola [*Actinidia deliciosa*]	136	24,392	19.3	3	0.599	Stress/defense/detoxification
gi|15637165|	Voltage-dependent anion channel [β *vulgaris*]	340	39,615	13.9	4	2.321	Transport
gi|225439482|	PREDICTED: importin subunit β-1 [*Vitis vinifera*]	65	90,956	3.9	2	2.014	Transport
gi| 526118004|	Probable E3 ubiquitin-protein ligase HERC1 [*Vitis vinifera*]	106	55,419	8	2	1.845	Transport
gi|147859669|	Hypothetical protein VITISV_026572 [*Vitis vinifera*]	105	32,300	9.1	2	1.988	Transport
gi|147842983|	Hypothetical protein VITISV_024360 [*Vitis vinifera*]	41	29,785	4	1	3.304	Transport
sp|Q41009|	Translocase of chloroplast 34 [*Pisum sativum*]	42	12,006	28.9	2	0.599	Transport
gi|87247471|	Putative glutathione *S*-transferase [*Populus x canadensis*]	295	31,506	16.9	2	0.577	Transport
gi|8896066|	FtsZ1 [*Tagetes erecta*]	71	30,227	11.2	2	2.163	Biological regulation and signal transduction
gi|71535005|	Zinc finger Glo3-like protein [*Medicago sativa*]	151	53,757	9.9	3	1.887	Biological regulation and signal transduction
sp|P93508|	Calreticulin [*Ricinus communis*]	250	22,819	30.5	4	1.79	Biological regulation and signal transduction
gi|255562771|	STS14 protein precursor, putative [*Ricinus communis*]	165	20,709	22.1	3	4.166	Biological regulation and signal transduction
gi|40807639|	Cystatin [*Actinidia eriantha*]	250	22,819	30.5	4	1.79	Biological regulation and signal transduction
gi|359497545|	PREDICTED: leucine-rich repeat receptor-like serine/threonine-protein kinase BAM1-like [*Vitis vinifera*]	250	22,819	30.5	4	1.79	Biological regulation and signal transduction
sp|Q8H100|	Probable ADP-ribosylation factor GTPase-activating protein AGD8 [*Spinacia oleracea*]	382	58,424	15.4	5	2.137	Biological regulation and signal transduction
gi|359495838|	PREDICTED: uncharacterized protein LOC100264206 [*Vitis vinifera*]	58	30,115	10.4	2	3.438	Biological regulation and signal transduction
sp|O23193|	CBS domain-containing protein CBSX1, chloroplastic [*Spinacia oleracea*]	138	27,995	20.6	3	1.672	Biological regulation and signal transduction
sp|P93654|	Syntaxin-22 [*Spinacia oleracea*]	35	30,780	8.7	2	0.522	Biological regulation and signal transduction
gi|350534900|	14-3-3 protein 3 [*Solanum lycopersicum*]	368	38,590	27.9	7	0.599	Biological regulation and signal transduction
gi|359492889|	PREDICTED: 14-3-3 protein [*Vitis vinifera*]	244	40,230	20.7	6	0.525	Biological regulation and signal transduction
sp|Q9FM65|	Fasciclin-like arabinogalactan protein 1 [*Spinacia oleracea*]	247	36,736	18.8	3	0.559	Biological regulation and signal transduction
gi|95116526|	Ethylene inducible protein hever [*Theobroma cacao*]	131	37,972	15.2	4	0.619	Biological regulation and signal transduction
sp|A1Y2B7|	Protein suppressor of gene SILENCING 3 homolog [*Zea mays*]	86	51,127	7.8	2	0.602	Biological regulation and signal transduction
gi|255587170|	Minichromosome maintenance protein, putative [*Ricinus communis*]	63	16,167	7.5	1	0.547	Biological regulation and signal transduction
gi|255571471|	Systemin receptor SR160 precursor, putative [*Ricinus communis*]	200	108,992	6.6	5	0.483	Biological regulation and signal transduction
gi|359494860|	PREDICTED: protein MOR1-like, partial [*Vitis vinifera*]	102	17,742	8.8	1	0.603	Biological regulation and signal transduction
gi|359495954|	PREDICTED: syntaxin-51-like [*Vitis vinifera*]	318	19,583	10.5	1	0.243	Biological regulation and signal transduction
sp|Q94KK7|	Syntaxin-52 [*Spinacia oleracea*]	40	31,732	6.3	1	0.594	Biological regulation and signal transduction
gi|359493650|	PREDICTED: early nodulin-like protein 2-like [*Vitis vinifera*]	214	26,770	23	4	0.538	Biological regulation and signal transduction
gi|225456479|	PREDICTED: signal recognition particle 68 kDa protein [*Vitis vinifera*]	52	30,059	4.6	1	0.623	Biological regulation and signal transduction
sp|O22126|	Fasciclin-like arabinogalactan protein 8 [*Spinacia oleracea*]	256	45,342	16.4	5	0.51	Biological regulation and signal transduction
gi|388491766|	Unknown [*Lotus japonicus*]	171	15,992	18.3	1	0.538	Biological regulation and signal transduction
gi|255545216|	Conserved hypothetical protein [*Ricinus communis*]	85	37,085	13	3	2.209	Unknown biological processes
gi|255547524|	Conserved hypothetical protein [*Ricinus communis*]	50	41,524	3.3	1	2.09	Unknown biological processes
sp|Q6YYB0|	UNCHARACTERIZED protein Os08g0359500 [*Oryza sativa* subsp*. Japonica*]	56	15,660	12.7	1	1.541	Unknown biological processes
gi|225423539|	PREDICTED: uncharacterized protein LOC100262861 [*Vitis vinifera*]	152	24,072	16	2	2.042	Unknown biological processes
gi|330318602|	Hypothetical protein [*Camellia sinensis*]	40	19,541	6	1	1.809	Unknown biological processes
gi|224070853|	Predicted protein [*Populus trichocarpa*]	104	23,465	22.3	3	1.788	Unknown biological processes
gi|224144195|	Predicted protein [*Populus trichocarpa*]	93	28,934	6.2	1	1.668	Unknown biological processes
gi|147818671|	Hypothetical protein VITISV_014852 [*Vitis vinifera*]	43	14,360	10.5	1	1.715	Unknown biological processes
gi|359488537|	PREDICTED: uncharacterized protein LOC100853981 [*Vitis vinifera*]	71	21,254	8.3	1	1.921	Unknown biological processes
gi|147818796|	Hypothetical protein VITISV_021596 [*Vitis vinifera*]	625	66,920	20.2	9	1.795	Unknown biological processes
gi|225452887|	PREDICTED: uncharacterized protein At5g39570 [*Vitis vinifera*]	141	28,611	31.3	4	1.902	Unknown biological processes
gi|359491847|	PREDICTED: uncharacterized protein LOC100240982 [*Vitis vinifera*]	78	22,451	15.1	2	1.893	Unknown biological processes
gi|225463725|	PREDICTED: uncharacterized protein LOC100261025 [*Vitis vinifera*]	79	82,239	7.5	3	1.632	Unknown biological processes
gi|147818796|	Hypothetical protein VITISV_021596 [*Vitis vinifera*]	625	66,920	20.2	9	1.795	Unknown biological processes
gi|356551464|	PREDICTED: uncharacterized protein LOC100807412 [*Glycine max*]	53	57,807	3	1	2.315	Unknown biological processes
gi|298205066|	Unnamed protein product [*Vitis vinifera*]	293	59,849	8	3	1.506	Unknown biological processes
gi|358249210|	Uncharacterized protein LOC100818758 [*Glycine max*]	74	26,221	15.1	2	2.26	Unknown biological processes
gi|224089721|	Predicted protein [*Populus trichocarpa*]	64	16,865	21	2	1.824	Unknown biological processes
gi|225443833|	PREDICTED: uncharacterized protein LOC100253185 [*Vitis vinifera*]	155	13,410	33	3	1.815	Unknown biological processes
gi|297743302|	Unnamed protein product [*Vitis vinifera*]	269	40,524	24.2	5	1.913	Unknown biological processes
gi|296081618|	Unnamed protein product [*Vitis vinifera*]	280	16,151	51.6	4	2.009	Unknown biological processes
gi|225459322|	PREDICTED: uncharacterized protein LOC100260886 isoform 2 [*Vitis vinifera*]	80	43,415	11.6	3	1.733	Unknown biological processes
sp|Q6ID70|	Uncharacterized protein At3g03773 [*Spinacia oleracea*]	189	28,352	12.6	2	1.772	Unknown biological processes
gi|225451915|	PREDICTED: uncharacterized protein LOC100244706 [*Vitis vinifera*]	181	28,654	12.2	2	2.492	Unknown biological processes
gi|297738842|	Unnamed protein product [*Vitis vinifera*]	68	67,341	4.9	2	0.657	Unknown biological processes
gi|359489218|	PREDICTED: uncharacterized protein LOC100232913 [*Vitis vinifera*]	95	31,024	10.9	2	0.565	Unknown biological processes
gi|17863981|	Unknown [*Davidia involucrata*]	150	95,611	9.6	6	0.598	Unknown biological processes
gi|224056457|	Predicted protein [*Populus trichocarpa*]	43	32,607	7.2	1	0.25	Unknown biological processes
gi|359488731|	PREDICTED: uncharacterized protein LOC100264617 [*Vitis vinifera*]	111	34,149	5.2	1	0.446	Unknown biological processes
gi|359476152|	PREDICTED: uncharacterized protein LOC100260975 [*Vitis vinifera*]	142	40,178	10.4	3	0.374	Unknown biological processes
gi|297742161|	Unnamed protein product [*Vitis vinifera*]	141	34,984	19.3	3	0.436	Unknown biological processes
gi|225449483|	PREDICTED: uncharacterized protein LOC100244410 [*Vitis vinifera*]	73	23,689	7.1	1	0.65	Unknown biological processes
gi|225463406|	PREDICTED: uncharacterized protein LOC100250442 [*Vitis vinifera*]	85	20,570	7.9	1	0.631	Unknown biological processes
gi|351722061|	Uncharacterized protein LOC100305495 precursor [*Glycine max*]	236	23,212	10.4	1	0.52	Unknown biological processes
gi|224141949|	Predicted protein [*Populus trichocarpa*]	101	18,222	19	1	0.514	Unknown biological processes

### 2.4. RT-qPCR Analysis and Enzyme Activity Assay

**Figure 4:**
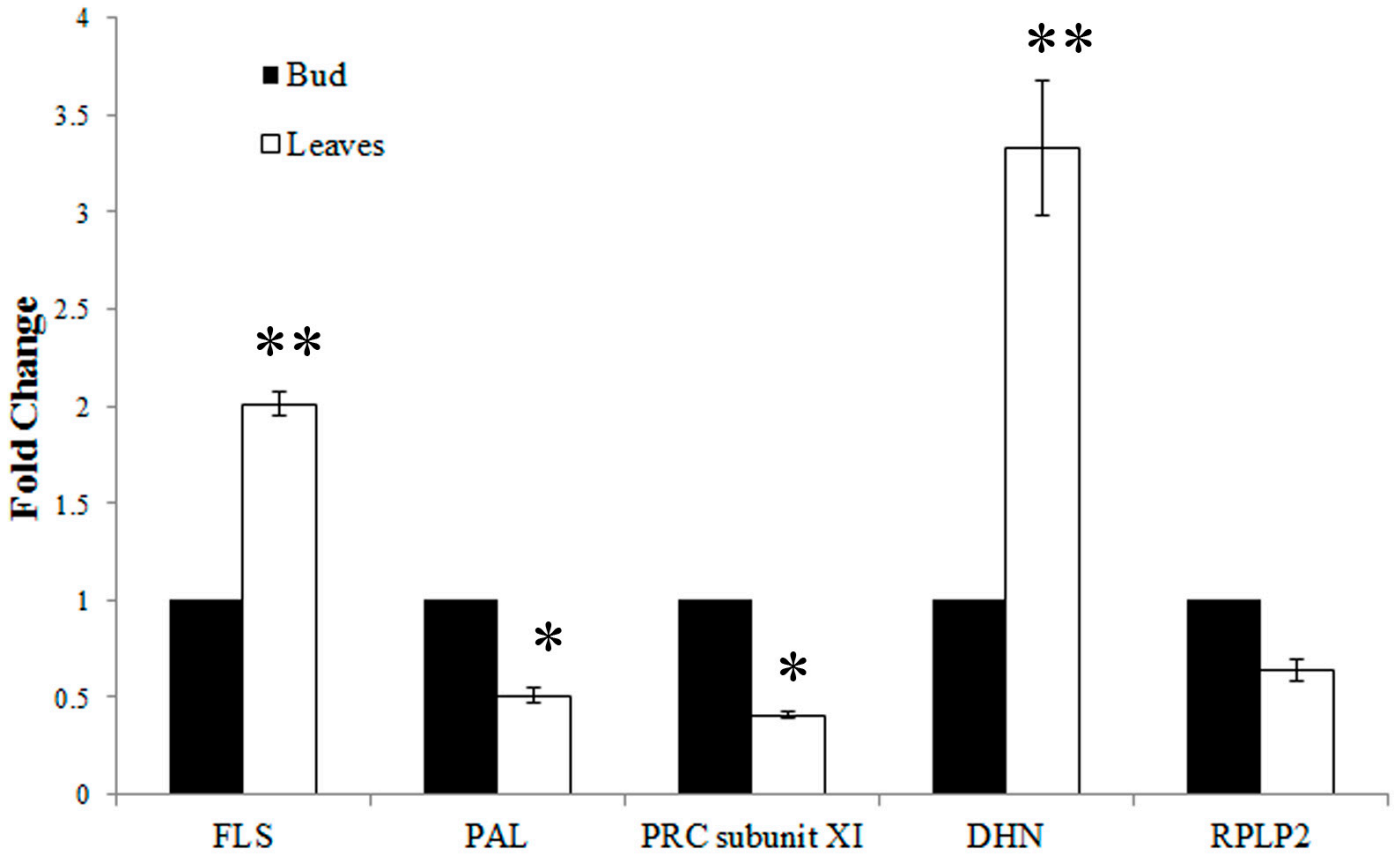
RT-qPCR analysis of the transcript levels of the differentially expressed proteins. FLS: flavonol synthase; PAL: phenylalanine ammonia-lyase; PRC subunit XI: photosystem I reaction center subunit XI; DHN: dehydrin; RPLP2: 60S acidic ribosomal protein p2. Statistical significance: * *p* < 0.05 and ** *p* < 0.01.

**Figure 5:**
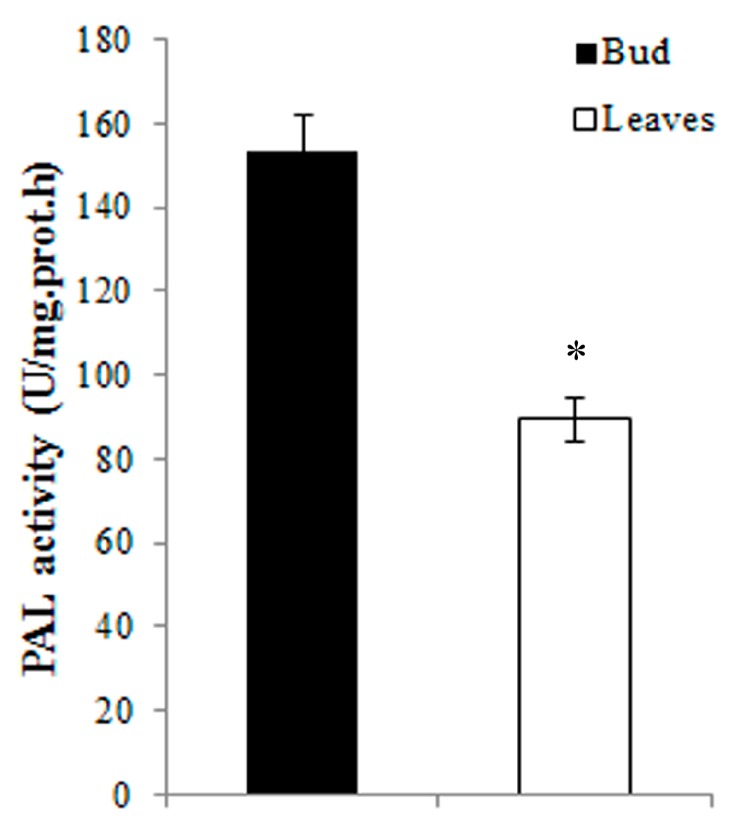
PAL activity in the buds and in young expanding leaves. Statistical significance: * *p* < 0.05.

To evaluate the iTRAQ results, RT-qPCR analysis and enzyme activity assays were performed. Five proteins were selected for RT-qPCR analysis; three were up-regulated (flavonol synthase, FLS; dehydrin, DHN; and 60S acidic ribosomal protein p2, RPLP2), and two were down-regulated (phenylalanine ammonia-lyase, PAL; photosystem I reaction center subunit XI, PRC subunit XI) in the young expanding leaves compared with the buds. As shown in Figure 4, the expression levels of FLS and DHN were significantly up-regulated in the young leaves compared with the buds (FLS: 2.01 ± 0.06-fold, *p* < 0.01. DHN: 3.33 ± 0.34-fold, *p* < 0.01). However, the expression levels of PAL and PRC subunit XI were significantly down-regulated in the young leaves compared with the buds (PAL: 0.51 ± 0.04-fold, *p* < 0.05. PRC subunit XI: 0.41 ± 0.02-fold, *p* < 0.05). The expression of RPLP2 was also down-regulated in the young expanding leaves compared with the buds (0.64 ± 0.05-fold), but no significant difference was observed (*p* > 0.05). The transcription levels of FLS, DHN, PAL and PRC subunit XI were closely correlated with the levels of their translation products in the buds and the young expanding leaves, whereas the RPLP2 transcript levels did not correspond with those of its translation products. As shown in Figure 5, PAL activity was significantly lower in young expanding leaves than in buds, which is consistent with its gene and protein expression levels in the buds and the young expanding leaves of tea plants.

## 3. Discussion

A previous study used subtractive cDNA library analysis to reveal the genes involved in the production of polyphenols and other secondary metabolites that are relatively abundant in young leaves [2]. However, because of post-transcriptional regulation, protein expression levels cannot always be predicted from quantitative mRNA data; the mRNA level does not always correlate with the protein level [10]. Therefore, proteomic analysis could improve our understanding of the molecular mechanisms underlying the change in the metabolite contents of the apical buds and the young expanding leaves of tea plants.

### 3.1. Changes in Secondary Metabolites

Tea leaves contain large amounts of flavonoids, including flavanones, flavones, flavonols, flavan-3-ols, and anthocyanidins. The predominant flavonoid in tea is catechin, which distinguishes tea from other plants and is an important determinant of tea quality and taste. A previous study showed that the concentrations of total catechins and polyphenols in tea leaves declined with leaf age, but changes in individual catechins varied [11]. Our HPLC analysis showed that EGCG and ECG were the most abundant catechins in both the buds and the young expanding leaves. These compounds exist in the green parts of tea seedlings but were not detected in the roots or cotyledons [12]. The catechins index [(EGCG + ECG)/EGC] was positively correlated with the sensory evaluation of brewed green tea [13]. Based on HPLC results, the green tea quality indexes of the buds and the young leaves were 45.11 and 15.59, respectively. These results were consistent with previous research [7,11]. Historically, tea has been valued for its purine alkaloids, including theobromine, theophylline and caffeine [14]. Theobromine is formed as part of the caffeine biosynthetic pathway and is produced in abundance if the methylation pathway of caffeine biosynthesis is absent [14]. An analysis of purine alkaloids in different tea seedling organs showed that more than 99% of the caffeine was in the leaves, with older leaves containing more per gram of fresh weight. Theobromine was found only in the younger leaves, and theophylline was either not present or present only in trace amounts [8]. Our study showed that the concentrations of theobromine and caffeine were lower in young expanding leaves, but no significant difference in theophylline levels was observed. Purine alkaloid metabolism also appears to be closely associated with leaf development and aging in tea seedlings [9,15]. The major biosynthetic route for caffeine is thought to be xanthosine→7-methyxanthosine→7-methylxanthine→theobromine →caffeine, and previous studies have indicated that caffeine biosynthesis was primarily controlled by the first *N*-methyl-transfer reaction, which is catalyzed by 7-methylxanthosine synthase [16,17]. Hence, the relatively lower caffeine and theobromine contents of young expanding leaves found in this study may be attributable either to a smaller supply of xanthosine for caffeine biosynthesis or to the lower activity of 7-methylxanthosine synthase in young expanding tea leaves.

### 3.2. Proteins Involved in Carbohydrate and Energy Metabolism

In plants, glycolysis and the tricarboxylic acid (TCA) cycle provide not only energy and cofactors but also important substrates for the synthesis of metabolites, as well as feedback signals [18]. Dynamic proteomic analysis revealed that the levels of glycolysis- and TCA cycle-related proteins increased during early-stage seed development in rice [19]. Our present results show that a subset of the differentially expressed proteins were involved in glycolysis and TCA, such as NADP-dependent glyceraldehyde-3-phosphate dehydrogenase (NADP-dependent GAPDH), dihydrolipoyl dehydrogenase (DLD), pyruvate dehydrogenase E3 subunit (PDE3), dihydrolipoamide succinyltransferase component of 2-oxoglutarate dehydrogenase (DLST) and phosphoenolpyruvate carboxylase (PEPC); these proteins were present at higher levels in the young, expanding leaves than in the buds. These results indicated that glycolysis and the TCA cycle increased in the young, expanding leaves and that more energy and substrates were required during the developmental stage at which young, expanding leaves are present.

### 3.3. Proteins Related to Secondary Metabolism

Polyphenols are the most important chemical compounds in tea plants, and have received increasing attention in recent years because of their benefits to human health [20,21,22,23]. The polyphenols in tea are predominantly members of three subclasses: flavanols, flavones and flavonols [24]. Four major catechins (flavanols), (−)-epicatechin (EC), (−)-epicatechin gallate (ECG), (−)-epigallocatechin (EGC), and (−)-epigallocatechin gallate (EGCG), constitute approximately one-third of the dry weight of green tea [25]. Quercetin, kaempferol, myricetin and their glycosides (flavonols), as well as apigenin glycosides (flavones), are also present, but at much lower concentrations [24]. Several proteins related to polyphenol biosynthesis were differentially expressed between the buds and the young, expanding leaves. Flavonol synthase (FLS), a dioxygenase that converts dihydroflavonols into flavonols, was initially found in parsley and was shown to require 2-oxoglutarare and Fe/ascorbate for full activity [26]. In FLS-silenced tobacco, there was a 25%–93% reduction in the flavonoid (quercetin) content and an increase in the catechin and epicatechin content [27,28]. Our previous study also indicated that FLS expression was a negative regulator of catechin biosynthesis, and especially of ECG and EGCG [29]. In our proteomic analysis, the expression of FLS was increased at the stage of young, expanding leaves, which indicated that at this stage, flavonol biosynthesis was enhanced and catechin biosynthesis was inhibited. These results also agree with our metabolic data, which show that compared with the buds, the flavonol content was greater and the total catechin content was lower in the young, expanding leaves. Isoflavone reductase homolog P3 belongs to the NmrA-type oxidoreductase family and the isoflavone reductase subfamily. Isoflavone reductase (IFR) specifically recognizes isoflavones and catalyzes a stereospecific, NADPH-dependent reduction to (3R)-isoflavanone [30]. In tea plants, IFR catalyzes the conversion of leucocyanidin and leucodelphinidin to (+)-catechin and (+)-gallocatechin, respectively. In our proteomic analysis, the expression of IFR homolog P3, which is involved in the accumulation of high levels of catechins, was more highly expressed in the buds compared with the young, expanding leaves. Phenylalanine ammonia-lyase (PAL) is an enzyme that catalyzes the conversion of l-phenylalanine to ammonia and *trans*-cinnamic acid [31]. PAL resides at a metabolically important position, linking secondary metabolism to primary metabolism. PAL is part of the first committed step in the phenylpropanoid pathway and is a key enzyme in the allocation of significant amounts of carbon from phenylalanine into the biosynthesis of several important secondary metabolites, such as lignins, flavonoids, and coumarins [32,33]. The overall flux into phenylpropanoid metabolism has been suggested to be regulated by PAL, which acts as a rate-limiting enzyme [34]. Park *et al.* found that PAL gene expression and catechin content were also reduced in mature leaves compared with young leaves [2]. A positive correlation between catechin content and the gene expression of PAL was observed under drought stress, after wounding and after abscisic acid treatment [35]. In the present study, the expression of both the PAL gene and protein were inhibited, and the catechin content was also reduced in young, expanding leaves. These results indicated that the carbon flux from phenylalanine into the biosynthesis of secondary metabolites was inhibited in the young, expanding leaves compared with the buds. Hydroxycinnamoyl-CoA: shikimate/quinate hydroxycinnamoyltransferase (HCT), which converts p-coumarate from CoA to shikimate/quinate esters, has been described as reversible enzyme [36]. It is involved in a step in lignin synthesis, and its down-regulation affects lignin content and composition [37,38]. In our proteomic analysis, the expression level of HCT was lower in the young, expanding leaves than in the buds. Arabidopsis plants in which HCT is silenced or lignin is repressed direct the metabolic flux into flavonoids through chalcone synthase [39], which may explain why the non-galloylated catechin content increased in the young, expanding tea leaves.

### 3.4. Photosynthetic Proteins

Photosynthesis is a key biological process in plant growth and development. In the present study, the abundance of several proteins involved in photosynthesis differed between the buds and the young expanding leaves. These proteins include ribulose-1,5-bisphosphate carboxylase/oxygenase (Rubisco) and its large subunit (RubiscoL), sedoheptulose-1,7-bisphosphatase (SBPase) precursor, photosystem I reaction center subunit XI (PS I-E), thylakoid lumenal 29 kDa protein (TL29), peroxiredoxin Q (PRXQ) and chlorophyll A/B binding protein (CitCAB1,2). Several studies have shown that during leaf development, photosynthetic activity gradually increases, and photosynthetic enzymes slowly accumulate [40,41,42,43]. Correlations between the photosynthetic rate and the catechin content of the leaves of tea plants showed that there was a positive correlation between the photosynthetic rate and the EC and GCG contents but a negative correlation between the photosynthetic rate, the total catechin content and the galloylated catechin content [44]. A study focusing on the relationship between the synthesis and accumulation of phenolics and flavonoids and the photosynthetic rate in ginger showed that when photosynthesis decreased, the synthesis of flavonoids such as quercetin, catechin, epicatechin and naringenin increased, and the soluble carbohydrates and plant biomass decreased [45]. The results of our proteomic analysis also showed that the expression of photosynthetic proteins was down-regulated in the buds compared with the young, expanding leaves. We infer that in the buds, the rate of photosynthesis is lower, so the carbon flow shifts from photosynthesis to the shikimic acid pathway, thereby producing more phenolics and flavonoids.

### 3.5. Defense-Related Proteins

The cellular antioxidant system consists of different enzymes. In our proteomic analysis, antioxidant proteins, such as superoxide dismutase (SOD), thioredoxin O2 (TO2), NADPH thioredoxin reductase (NADPH-TR), and glutaredoxin (GRX), were more abundant in young, expanding leaves than in buds. The activity of antioxidant enzymes, such as SOD also increased at early stages of leaf expansion and was sustained throughout leaf expansion [46,47]. Therefore, the proteins involved in the antioxidant system may be related to leaf expansion. Another study also indicated that a certain concentration of reactive oxygen species (ROS) is necessary for leaf elongation, but it could not be determined if H_2_O_2_ or other ROS are the active agents [48]. We suggest that the accumulation of antioxidant proteins could dissipate excess excitation energy and protect leaves against photodamage, which can be caused by a certain levels of ROS in expanding tea leaves.

## 4. Experimental Section

### 4.1. Plant Materials

**Figure 6:**
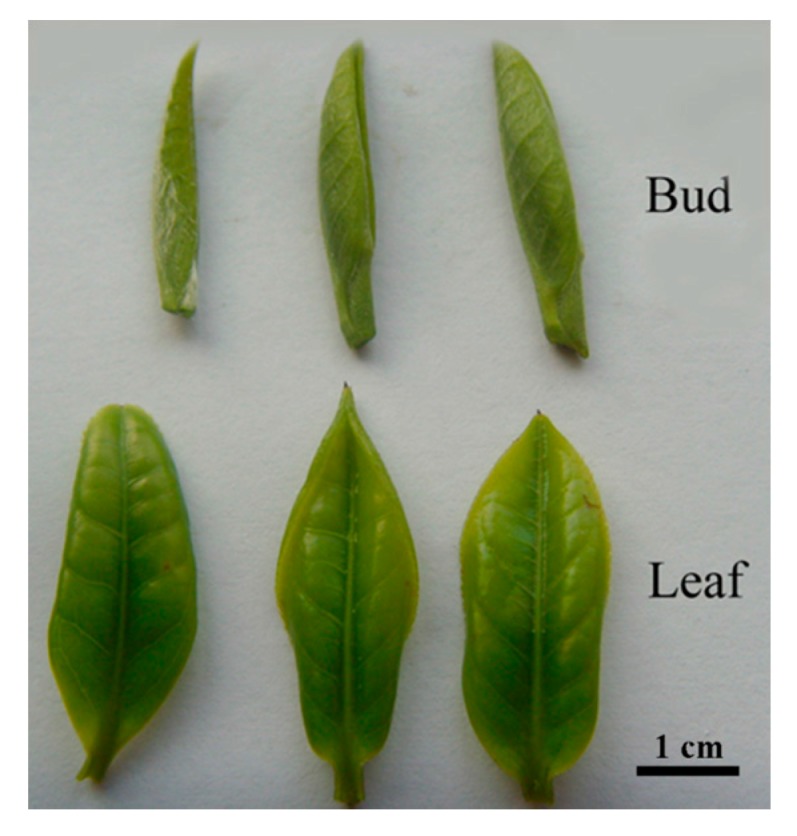
The buds and young expanding leaves of tea plants.

Tea plants were grown in the experimental tea garden of Hunan Agricultural University in Changsha, China. The apical buds and the first unfolding leaves were plucked from the same plants at different stages of development, briefly washed with sterile water, immediately frozen in liquid nitrogen and stored at −80 °C prior to analysis (Figure 6).

### 4.2. Metabolic Analysis of Tea Samples

Total polyphenols, catechins and alkaloids were extracted from the samples and analyzed as previously described with a slight modification [29]; a total of 0.20 g of freeze-dried, ground leaves was accurately weighed and extracted twice with 5 mL of a 75:25 (*v*/*v*) ethanol:water solution at 80 °C for 15 min. The extract was filtered through filter paper and then diluted to 50 mL. The total polyphenol and flavonoid content in the sample was determined using the ferrous tartrate method [49] and the aluminum trichloride method [50]. The catechin and alkaloid contents were determined with high-performance liquid chromatography (HPLC) according to Wang *et al.* [51] with slight modifications. A Shimadzu HPLC system (Shimadzu, Tokyo, Japan) with 10AD dual pumps was used with a reversed-phase column (Welchorm C18 200 × 4.6, 5 μm), a mobile phase of distilled water (A) and a mobile phase (B) of 40% *N*,*N*-dimethylformamide, 2% methanol and 1.5% acetic acid. The gradient was as follows: 0.01–13.00 min, linear gradient from 14% to 23% B; 13.00–25.00 min, linear gradient from 23% to 36% B; 25.00–28.00 min, 36% B; 28.00–30.00 min, linear gradient from 36%–14% B; 30.00–34.00 min, 14% B. The samples were eluted at 35 °C and at a flow rate of 1.00 mL/min. The chromatograms were recorded at 278 nm. The peaks were identified by comparing the retention times of the sample to those of authentic standards. The extraction for flavone hydrolysis was carried out as follows: plant material (0.5 g dry weight) was mixed with 20 mL methanol and 2.0 mL HCl (6 M). After refluxing at 95 °C for 1.5 h, the hydrolyzed solution was filtered through filter paper, then diluted to 25 mL with methanol. Flavonols were detected with the following HPLC method [52]: the mobile phase consisted of 30% acetonitrile in 0.025 M KH_2_PO_4_ buffer solution (*v*/*v*); the pH of the mobile phase was adjusted to 2.5 using H_3_PO_4_. The samples were eluted at 35 °C at a flow rate of 1.00 mL/min and were monitored at 370 nm. The peaks were identified by comparing the retention times of the sample to those of authentic standards. All experiments included three separate biological replicates.

### 4.3. Protein Extraction

Leaf samples were weighed and ground in liquid nitrogen, then suspended in lysis buffer [7 M urea, 2 M thiourea, 4% 3-[(3-Cholamidopropyl)dimethylammonio]propanesulfonate (CHAPS), 40 mM *Tris*-HCl, pH 8.5, 1 mM Phenylmethanesulfonyl fluoride (PMSF), 2 mM Ethylene Diamine Tetraacetic Acid (EDTA), 10 mM dl-Dithiothreitol (DTT)] and kept in an ice bath for 2 h. After this 2 h lysis, the samples were sonicated in an ice bath for 15 min and were clarified by centrifugation at 25,000× *g*. The supernatant was collected, and the protein concentration was determined with a 2D quantification kit (GE Healthcare, Chalfont St. Giles, Buckinghamshire, UK).

### 4.4. iTRAQ Analysis

iTRAQ analysis was performed at the Beijing Genomics Institute (BGI, Shenzhen, China). Protein samples were reduced with 10 mM DTT, alkylated with 55 mM iodoacetamide, digested using sequencing-grade trypsin (Promega, Madison, WI, USA), and labeled using an iTRAQ Reagent Multiplex Kit (AB SCIEX, Foster City, CA, USA) according to the manufacturer’s protocol. The bud and leaf samples were labeled with 114 and 117 Da, respectively. After labeling, all samples were pooled and purified using a strong cation exchange chromatography (SCX) column (Phenomenex, Torrance, CA, USA) with an LC-20AB HPLC system (Shimadzu, Tokyo, Japan). The labeled peptides were separated with mobile phase B (2% water, 98% acetonitrile and 0.1% formic acid) at a flow rate of 300 nL/min, 0%–5% over 1 min, 5%–35% over 40 min and 35%–80% over 5 min on a nanoACQuity system (Waters, Milford, MA, USA). The LC fractions were analyzed using a Triple TOF 5600 mass spectrometer (AB SCIEX, Foster City, CA, USA) fitted with a Nanospray Ⅲ source (AB SCIEX, Concord, MA, USA) and a pulled quartz tip (New Objectives, Woburn, MA, USA). The data were acquired using an ion spray voltage of 2.5 kV and an interface heater temperature of 150 °C. Curtain gas and nebulizer gas were delivered at 30 pounds per square inch (PSI) and 15 PSI, respectively. For information-dependent acquisition (IDA), survey scans were acquired in 250 ms, and once the detection of ions with a 2+ to 5+ charge state crossed a threshold of 150 counts per second, as many as 35 product ion scans were collected. The total cycle time was fixed at 2.5 s. A rolling collision energy setting was applied to all precursor ions for collision-induced dissociation (CID). Two independent biological experiments with three technical replicates each were performed.

### 4.5. Data Analysis

MS/MS data acquisition was performed with Analyst QS 2.0 software (AB SCIEX, Foster City, CA, USA). For protein identification, MS/MS data were searched against the “plant” subset of the National Center for Biotechnology Information Non-redundant protein sequences (NCBInr) database using Mascot version 2.3.02 (Matrix Science, London, UK). The search parameters were as follows: a peptide mass tolerance of 10 ppm was allowed for intact peptides and ± 0.05 Da for fragmented ions; a maximum of one missed cleavage was allowed in the trypsin digests; cysteine carbamidomethylation was considered a fixed modification; glutamine pyrophosphorylation variable oxidation of methionine and iTRAQ labeling of tyrosine were set as variable modifications; carbamidomethylation of cysteine and iTRAQ labeling of lysines and the N-terminal amino group of peptides were set as fixed modifications. Only peptides with significance scores greater than “identity score” were considered identified, and a protein was considered identified if at least one such unique peptide match was apparent for the protein. For protein quantitation, the peptide to be quantified was automatically selected using the Pro Group algorithm to calculate the reporter peak area, the error factor (EF), and the *p*-value. Proteins with a fold change of >1.5 and a two-tailed *p*-value of less than 0.05 were considered to have significantly different expression.

### 4.6. Bioinformatic Analysis of Proteins

Differentially expressed proteins were mapped to Gene Ontology Terms (GO) using a local Bell Labs Layered Space-Time (BLAST) against a reference database downloaded from the website (GO-Annotation@EBI). The Clusters of Orthologous Groups of Proteins system (COG) can be used to functionally annotate genes from new genomes and for research on genome evolution [53]. The Kyoto Encyclopedia of Genes and Genomes (KEGG) is an updated system that computerizes current knowledge on biochemical pathways and other types of molecular interactions and can be used as a reference for the systematic interpretation of sequencing data [54]. To augment the biological and functional properties of differentially expressed proteins, the proteins were further analyzed using the COG (http://www.ncbi.nlm.nih.gov/COG/) and KEGG databases (http://www. genome.jp/kegg/pathway.html).

### 4.7. Real-time Quantitative PCR Analysis

Total RNA for RT-qPCR analysis was extracted from leaves at the two developmental stages using an RNeasy Plant Mini Kit (Qiagen, Hilden, Germany) and an RNase-Free DNase Set (Qiagen, Hilden, Germany). cDNA was synthesized from the total RNA (1 μg) using oligo(dT)18 primers and Moloney murine leukemia virus reverse transcriptase (Promega, Madison, WI, USA) according to the manufacturer’s instructions. The primers used for RT-qPCR (Table 2) were designed using Beacon Designer 7.0 software (Premier Biosoft, Palo Alto, CA, USA) and were based on the cDNA sequences. The reactions were carried out with a Rotor-Gene Q 6200 real-time PCR system (Qiagen, Hilden, Germany) using three-step cycling conditions of 95 °C for 10 min followed by 45 cycles of 95 °C for 10 s, 56 °C for 15 s and 72 °C for 20 s. The reaction mixture (20 μL) contained 1 μL of cDNA solution, 10 μL of Platinum SYBR^®^ Green qPCR SuperMix-UDG (Invitrogen, Carlsbad, CA, USA) and primers at a concentration of 6 μM each. For each RT-qPCR sample, there were three biological replicates with three technical replicates. The GAPDH gene was used as an internal standard for the normalization of gene expression, and the tea buds were used as a reference sample whose value was set to 1. The relative gene expression was evaluated using the comparative cycle threshold method [55].

**Table 2:** Primers used in RT-qPCR analysis.

Gene Name	Accession Number	Primer Sequence (5′–3′)
Flavonol synthase	DQ198089	Forward: ggagaacagcaaggctatcg
		Reverse: tctcctcctgtgggagctta
Phenylalanine ammonia-lyase	D26596	Forward: tccgatcatcgacaaaatca
		Reverse: agctcagagaattgggcaaa
Photosystem I reaction center subunit XI	HM003371	Forward: tcaaagaaggagagccatcg
		Reverse: gcaagaaataggcccaaatg
Dehydrin	FJ436978	Forward: gaggagaggaccaacagcag
		Reverse: acgacaccgacacacacatt
60S acidic ribosomal protein p2	HM003314	Forward: gggtgctattgcagtgacct
		Reverse: attgggggagaaagaaggaa

### 4.8. PAL Extraction and Enzyme Assays

Tea samples (1 g) were ground into a fine powder with a mortar and pestle in liquid N_2_. The powder was extracted with 5 mL of extraction buffer 50 mM Tris-HCl pH 8.9, 10 μM leupeptin, 5 mM EDTA, 15 mM β-mercaptoethanol, 5 mM Vc, 1 mM PMSF, 0.15% Polyvinylpyrrolidone (PVP)], and then was stirred on ice for 10 min. Subsequently, the mixture was centrifuged at 30,000× *g* for 30 min at 20 °C. The supernatant was stirred on Dowex (1 × 2) in the chloride (Cl) form for 30 min to remove residual phenolics. The cleared supernatant was used in a PAL enzyme assay. The protein concentrations in the enzyme extract were measured with a 2D quantification kit (GE Healthcare, Chalfont St Giles, Buckinghamshire, UK). PAL activity was assayed using the method of Solecka and Kacperska [29].

### 4.9. Statistical Analysis

Statistical analyses were performed using the Statistical Package for the Social Sciences software (SPSS; Chicago, IL, USA). ANOVA and Student’s *t*-tests were used to determine significant differences between different groups. A *p*-value <0.05 was considered significant.

## 5. Conclusions

The quantitative protein expression data presented in this study provide a global overview of a set of proteins that are expressed in the buds and the young, expanding leaves of tea. A total of 233 proteins were identified as being differentially expressed between the buds and the young leaves. A large array of diverse functions, including energy metabolism and the metabolism of carbohydrates, secondary metabolites, nucleic acids and proteins, as well as photosynthesis and defense-related processes, were revealed. Based on these results, we infer that the proteins involved in polyphenol biosynthesis and photosynthesis may also mediate the secondary metabolite content in tea plants. The proteins related to energy and antioxidant metabolism may promote tea leaf development. However, the RT-qPCR results showed that the protein expression levels did not closely correlate with their gene expression levels. Overall, these findings improve our understanding of the molecular mechanisms underlying the change in the metabolite content from the buds to the young, expanding leaves of tea plants.

## Supplementary Materials

Supplementary materials can be found at http://www.mdpi.com/1422-0067/16/06/14007/s1.
