# Supplementary material for: A Comparative Proteomic Analysis of the Buds and the Young Expanding Leaves of the Tea Plant (Camellia sinensis L.)
Source: Int J Mol Sci. 2015 Jun 18;16(6):14007–38. doi: 10.3390/ijms160614007 (PMC4490536; doi:10.3390/ijms160614007)
Supplement: Supplementary file 1 [file ijms-16-14007-s001.pdf]

Supplementary Information

Table S1. Gene ontology analysis.

Gene Ontology Term	Cluster Frequency	Protein Frequency of Use	*p*-Value
Ribonucleoprotein complex	27 out of 162 genes, 16.7%	135 out of 1663 genes, 8.1%	0.000118
Endoplasmic reticulum	5 out of 162 genes, 3.1%	20 out of 1663 genes, 1.2%	0.038199
Endoplasmic reticulum part	5 out of 162 genes, 3.1%	20 out of 1663 genes, 1.2%	0.038199

Table S2. KEGG pathway analysis.

Pathway	Different Proteins with Pathway Annotation (165)	All Proteins with Pathway Annotation (1756)	*p*-Value	Pathway ID
Ribosome	26 (15.76%)	98 (5.58%)	3.15×10^{-7}	ko03010
Sulfur metabolism	3 (1.82%)	7 (0.4%)	0.02147144	ko00920
Phenylalanine metabolism	6 (3.64%)	26 (1.48%)	0.02949184	ko00360
SNARE interactions in vesicular transport	3 (1.82%)	9 (0.51%)	0.04477584	ko04130
